# Temporoparietal fascial flap for primary reconstruction of complex ring avulsion injury: A case report

**DOI:** 10.1016/j.jpra.2022.04.002

**Published:** 2022-04-29

**Authors:** Shane Cullen, Kenneth Joyce, Christoph Theopold

**Affiliations:** Dept of Plastic and Reconstructive Surgery, St James’ Hospital, James's St, Dublin 8, Dublin D08 NHY1, Ireland

**Keywords:** Ring avulsion, Degloving, Finger amputation, Temporoparietal flap

## Abstract

Ring avulsion injuries pose difficult treatment decisions for hand surgeons. Urbaniak class III injuries have poor replantation rates, therefore other reconstructive options have to be explored. We present the case of a 39 year old fireman with a complete ring avulsion injury of the left ring finger that was reconstructed with a free temporoparietal fascial flap, covered with a split skin graft. At 6 months follow-up the patient had an excellent range of motion with acceptable aesthetic result but no sensation.

## Introduction

Ring avulsion injuries have been classified by Urbaniak and subsequently Adani, based on the severity of vascular, bone and joint injury.^1^^,^^2^ The poorest prognosis is for those classified as type III injuries where a bony injury is combined with both arterial and venous insufficiency. Avulsion injuries are commonly cited as having poor functional outcomes after replantation.^2^ Therefore, when the amputated part is not suitable for replantation, favoured reconstructive options in the literature include fasciocutaneous or adipofascial flaps, including groin flaps. Groin flaps tend to result in bulky fingers with mobile tissue and can have a negative impact on tendon glide and functional outcomes. To date there are only two cases reported of temporoparietal fascial (TPF) flaps used for reconstruction of complete ring avulsion injuries, with the last being reported over 20 years ago.^3^^,^^4^ In both cases, no specific parameters of functional outcome were reported. Hyperpigmentation of the reconstructed finger was reported. We report one such case, highlighting its merits as an excellent flap for reconstruction of ring avulsion injuries.

## Case report

**Figure 1:**
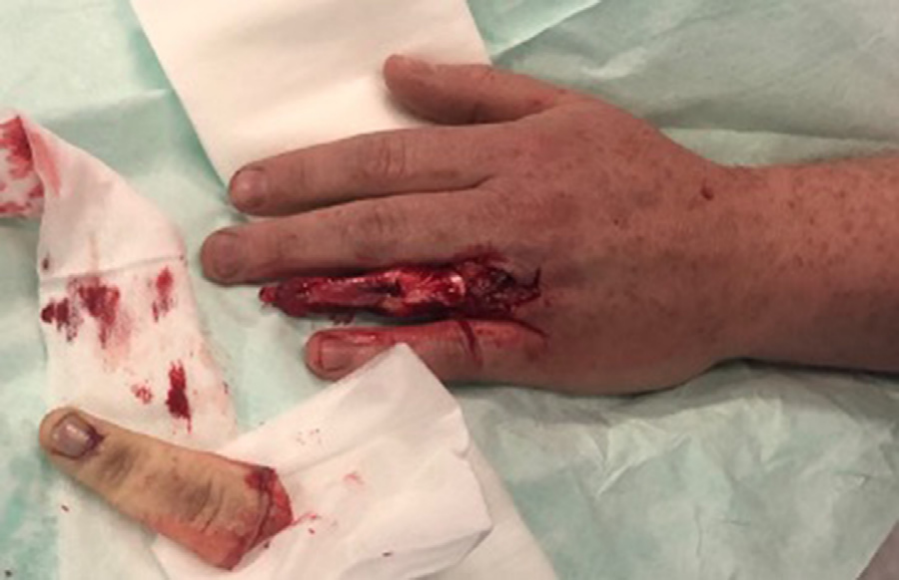
Initial presentation with Finger Avulsion Urbaniak III injury.

A 39-year-old, right hand dominant, fireman presented with a complete traumatic avulsion of the left ring fasciocutaneous envelope and nail apparatus. The injury occurred when his wedding ring got caught on a spike whilst climbing a fence. There was complete degloving of the soft tissues of the finger, including the nail apparatus, pulp and a small portion of the distal phalanx, distal to the waist, with exposed flexor and extensor tendons (Urbaniak class III) [Figure 1]. Because the neurovascular bundles had been avulsed from the skin envelope quite distally and were thrombosed over a long distance, it was deemed unlikely that primary replantation, even with vein interposition grafts, would result in a viable replant. The patient preferred not to undergo primary amputation and consented to reconstruction with a temporoparietal fascial (TPF) flap.

A TPF flap was raised through a “Y” incision extending from the left pre-auricular area over the parietal scalp. An 8 × 10 cm segment of temporoparietal fascia was dissected and elevated on the superficial temporal artery and vein. The temporal branch of the facial nerve was identified and protected. The artery and vein were separated over approximately 4 cm, to permit end-to-end microvascular anastomoses to the digital artery and a dorsal vein at the level of the metacarpophalangeal joint.

**Figure 2:**
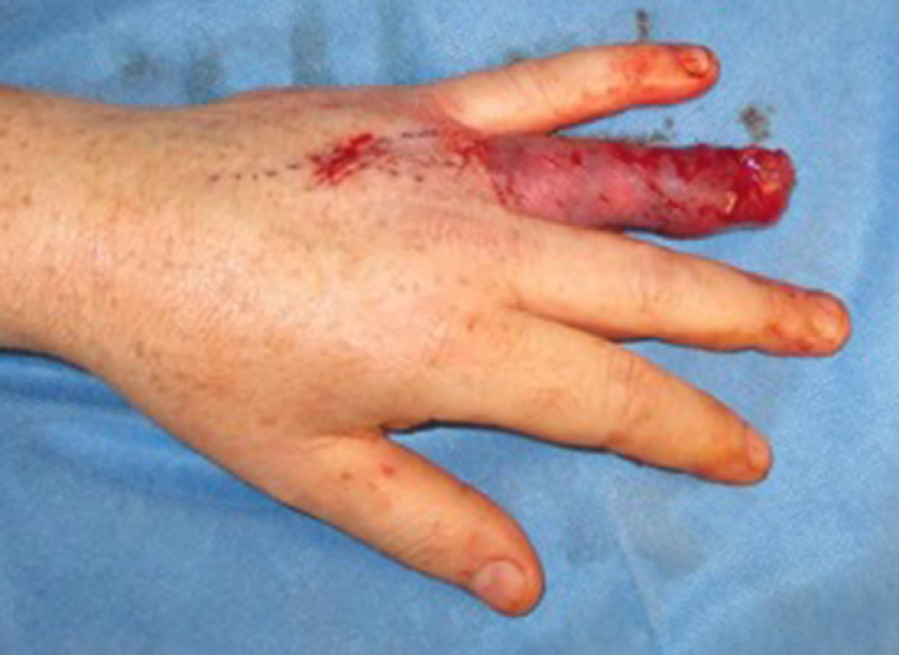
TPF flap inset and split skin graft.

The digital nerves were avulsed during the injury and were not reconstructed. The TPF flap was wrapped around the ring finger and secured proximally and distally and covered with a split thickness skin graft from the thigh (Figure 2). The patient's post-operative course was uneventful and he was discharged day two post-operatively. Graft check at day seven post-op showed good adherence. The patient was immobilized in a thermoplastic split until three weeks post-op. At this stage his graft was felt to be robust enough to tolerate active and passive movement. Figure six and Video 1 (Supplementary material) show the finger appearance at six weeks post-op. Table 1 demonstrates his hand function at six weeks and six months following reconstruction.

**Table 1:** Functional Outcomes at Six weeks and Six Months.

Outcomes	6 Weeks Post-op	6 months Post-op
Range of Motion (degrees)		
MCPJ	60	85
PIPJ	65	90
DIPJ	20	45
Grip Strength (kg)		
Right hand	410N	430N
Left Hand	200N	340N
% Comparison	49%	79%
2-Point Discrimination	Insensate	Insensate

Finger function outcomes following reconstruction of left ring finger. [MCPJ = metacarpophalangeal joint, PIPJ = proximal interphalangeal joint, DIPJ = distal interphalangeal joint]

**Figure 3:**
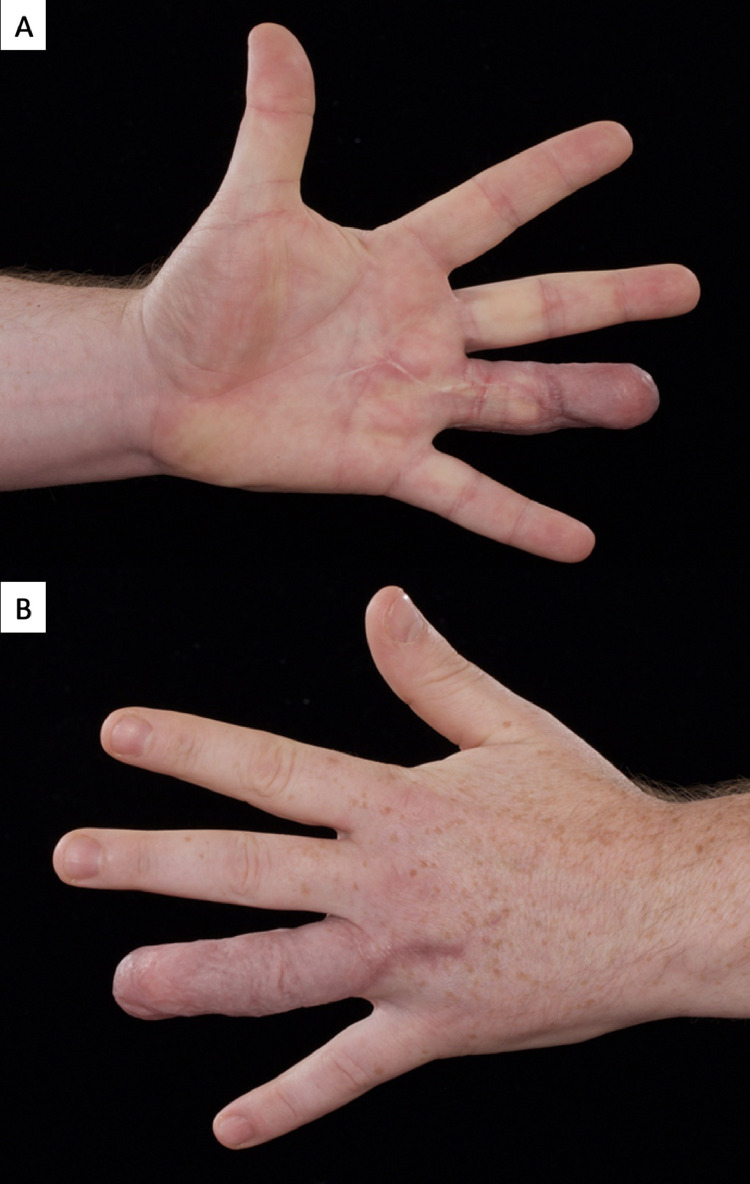
A. Volar surface of hand with ring finger TPF flap at 6 months post-op. B. Dorsum of hand with TPF flap at 6 months post-op.

The patient continues to progress with hand therapy. He is pleased with the aesthetic appearance and denies any cold intolerance. He reports intermittent swelling of the digit which resolves with elevation and compression dressings. He has returned to driving his car. Maximal grip strength at 6 months post-injury was 340 N, representing 80% of the force achieved by his uninjured, dominant hand. This has been deemed sufficient for a return to full duties as a fireman Figure 3.

## Discussion

Mechanism of injury in finger amputations is one of the key prognostic factors in determining potential for replantation. In ring avulsion injuries digital arteries are usually damaged at multiple levels and stretched vessels carry a high risk of intimal damage. Urbaniak advocated for revision amputation to treat complete finger avulsion injuries.^1^ A systematic review reported a mean survival rate for complete finger and thumb avulsions undergoing replantation was 66% compared to 87% in clean cut injuries, indicating the severity of tissue trauma caused by the traction force that occurs.^5^

Successful ring avulsion reconstructions depend on patient selection. Coverage of exposed tendons or bones at the dorsal and palmar surface of the finger requires thin, supple tissue to provide glide for adequate range of motion and a satisfactory aesthetic result.^6^ Many fasciocutaneous flaps have been described for defect coverage in the hand when free tissue transfer is indicated.^7^ The main disadvantage of many flaps is their bulk, caused by a thick subcutaneous layer, and the frequently unsightly donor site.^6^

Traditionally the most common reconstructive option for finger degloving injuries is a fasciocutaneous flap, such as a tubed groin flap (either pedicled or as a free flap). Their inherent bulk can significantly reduce finger flexion, thus requiring secondary revision to thin the flap.^8^ Laminated skin grafted fascial flaps (such as the TPF flap) are often preferred to bulkier fasciocutaneous flaps due to the thin and pliable characteristics of these.

The free TPF flap was initially described as a fascial scalp flap by Smith in 1980 [6]. It is thin, pliable, well-vascularized tissue that allows free gliding of tendons and easy mobilization of joints. The TPF flap allows overlying skin grafts to adhere well, provides an acceptable contour, and has proven to be durable over time.^9^ It is quick to harvest, has a pedicle sufficiently long to reach arteries and veins in the carpus, and permits a two-team approach. The donor site is hidden in the hair bearing scalp, giving a cosmetically satisfactory result with no functional deficit. The TPF flap can be reliably used as a sensate pedicled or free fasciocutaneous flap when auriculotemporal nerve is preserved and elevated with the flap.^10^

Given the TPF flap has numerous characteristics which make it an ideal reconstructive option for complex ring avulsion injuries despite its rare utilisation. Venous insufficiency is more commonly reported than arterial insufficiency.^10^ This problem can be prevented by preserving more soft tissue around the pedicle, avoiding kinking of the pedicle, as well as performing the anastomoses outside the zone of injury. Regarding donor site morbidity, conspicuous widened scars or alopecia scars in short-haired patients can be a relative disadvantage, however the risk of such complications are low.^10^ Hyperpigmentation of the overlying skin graft has also been reported though causes minimal patient dissatisfaction.^6^

In conclusion, the temporoparietal free fascial flap provides a useful reconstructive option for the soft tissue envelope of the finger following ring avulsion injury when the avulsed tissue is deemed unfit for replantation. Its thickness, pliability, high vascularity, with minimal donor site morbidity, provides glide for adequate range of motion. Excellent functional and cosmetic outcomes should favour its preferential reconstructive use in such injuries.

## Contributor ship

Nil.

## Funding

The author(s) received no financial support for the research, authorship, and/or publication of this article.

## Ethical approval

St. James's University Hospital, Dublin does not require ethical approval for reporting individual case reports.

## Informed consent

Written informed consent was obtained from the patient(s) for their anonymized information to be published in this article.

## Declaration of Competing Interest

The author(s) declare no potential conflicts of interest with respect to the research, authorship, and/or publication of this article.

## Appendix Supplementary materials

Supplemental Material: Hand range of movement taken at six months post-op. Functional Outcomes at Six weeks and Six Months. Image, video 1
